# Taming the massive genome of Scots pine with PiSy50k, a new genotyping array for conifer research

**DOI:** 10.1111/tpj.15628

**Published:** 2022-01-16

**Authors:** Chedly Kastally, Alina K. Niskanen, Annika Perry, Sonja T. Kujala, Komlan Avia, Sandra Cervantes, Matti Haapanen, Robert Kesälahti, Timo A. Kumpula, Tiina M. Mattila, Dario I. Ojeda, Jaakko S. Tyrmi, Witold Wachowiak, Stephen Cavers, Katri Kärkkäinen, Outi Savolainen, Tanja Pyhäjärvi

**Affiliations:** ^1^ Department of Ecology and Genetics University of Oulu P.O. Box 3000 90014 Oulu Finland; ^2^ UK Centre for Ecology & Hydrology Bush Estate Penicuik Midlothian EH26 0QB UK; ^3^ Natural Resources Institute Finland (Luke) Paavo Havaksen tie 3 90570 Oulu Finland; ^4^ Université de Strasbourg INRAE SVQV UMR‐A 1131 F‐68000 Colmar France; ^5^ Natural Resources Institute Finland (Luke) Latokartanonkaari 9 FI‐00790 Helsinki Finland; ^6^ Department of Organismal Biology EBC Uppsala University Norbyvägen 18 A Uppsala 752 36 Sweden; ^7^ Norwegian Institute of Bioeconomy Research P.O. Box 115 Ås 1431 Norway; ^8^ Institute of Environmental Biology Faculty of Biology Adam Mickiewicz University in Poznań Uniwersytetu Poznańskiego 6 61‐614 Poznań Poland; ^9^ Department of Forest Sciences University of Helsinki P.O. Box 27 00014 Helsinki Finland

**Keywords:** *Pinus sylvestris*, genotyping, pedigree, single‐nucleotide polymorphism, genetic diversity

## Abstract

*Pinus sylvestris* (Scots pine) is the most widespread coniferous tree in the boreal forests of Eurasia, with major economic and ecological importance. However, its large and repetitive genome presents a challenge for conducting genome‐wide analyses such as association studies, genetic mapping and genomic selection. We present a new 50K single‐nucleotide polymorphism (SNP) genotyping array for Scots pine research, breeding and other applications. To select the SNP set, we first genotyped 480 Scots pine samples on a 407 540 SNP screening array and identified 47 712 high‐quality SNPs for the final array (called ‘PiSy50k’). Here, we provide details of the design and testing, as well as allele frequency estimates from the discovery panel, functional annotation, tissue‐specific expression patterns and expression level information for the SNPs or corresponding genes, when available. We validated the performance of the PiSy50k array using samples from Finland and Scotland. Overall, 39 678 (83.2%) SNPs showed low error rates (mean = 0.9%). Relatedness estimates based on array genotypes were consistent with the expected pedigrees, and the level of Mendelian error was negligible. In addition, array genotypes successfully discriminate between Scots pine populations of Finnish and Scottish origins. The PiSy50k SNP array will be a valuable tool for a wide variety of future genetic studies and forestry applications.

## Significance Statement

In the genomic era, the gigantic size of conifer genomes still hampers advances in a wide range of fields of applied and fundamental sciences. To facilitate Scots pine genetic research and modern breeding methods, we developed a 50K SNP genotyping array and provide information that links the array markers to genetic diversity and gene expression levels across tissues.

## INTRODUCTION

To understand the genotype–phenotype link in plants, tools connecting phenotype, genotype, gene expression and environment are needed. Many forest trees and especially gymnosperms have a long history of studies on the phenotypic variation across geographical regions and environmental conditions (Alberto et al., 2013). However, much of this knowledge is disconnected from molecular biology and genetic variation as genomic resources have mainly been developed in and for short‐lived herbaceous plants or broadleaved trees, but are still lacking for gymnosperms (Isabel et al., 2020; Wegrzyn et al., 2020). Fast and affordable genotyping tools, accompanied with knowledge on gene expression, annotation and population allele frequencies, provide a link between the wealth of gymnosperm phenotypic data with molecular genetic mechanisms and the effects of natural selection. Such tools for gymnosperms are especially valuable as they could inform us about the evolution of phenotypes that appeared independently in both angiosperms and gymnosperms. In addition to connecting genomic and phenotypic resources, a better understanding of the evolution of major adaptations in trees would be of great value in tree breeding and in forestry, a major player in the bioeconomy and carbon sequestration.

*Pinus sylvestris* (Scots pine) is one of the world’s most widely distributed conifers (Houston Durrant et al., 2016) and is dominant in forests across 145 million hectares in Northern Eurasia (Mason and Alía, 2000; Mullin et al., 2011; Pyhäjärvi et al., 2020). Scots pine is an essential part of boreal forests, which are significant carbon sinks (Pan et al., 2011). It is also an important source of timber and other wood‐based products (CABI, 2013). In some areas, there is currently substantial interest in further genetic improvement of the species to reduce national dependency on exotic trees.

Breeding activities of Scots pine are mostly conducted in Fennoscandia and the Baltic region, with Sweden and Finland conducting the most advanced breeding programs (Haapanen et al., 2015). Compared with unimproved trees, genotypes selected based on progeny testing are expected to provide gains of 20–25% per unit area of wood production in seeds/seedlings produced in open‐pollinated seed orchards (Haapanen et al., 2016; Jansson et al., 2017; Rosvall et al., 2001). These improvements have significant implications on both economic perspectives and future carbon sequestration capacities. Adding genomics to the breeding scheme can further advance future gains. Forest tree breeding programs traditionally operate on large numbers of individuals. Cost‐effective genotyping platforms are therefore essential for incorporating genomics into tree breeding schemes to the extent now true for cattle and crop breeding (Grattapaglia et al., 2018; Isik, 2014; Meuwissen et al., 2016; Voss‐Fels et al., 2019).

Similarly to most gymnosperms, however, the large genome size of the Scots pine (24 Gbp, up to 35 Gb in *Pinus gerardiana*; Zonneveld, 2012) has restricted the use of genomic approaches, such as whole‐genome sequencing (WGS) for the identification of functionally important variation in genome‐wide association studies (GWAS). Genotyping 500 individuals, a fairly low sample size for GWAS, with 20× coverage would require a prohibitive volume of 240 Tbp of sequencing data and considerable investments in computing resources and time (Wang et al., 2020). Moreover, most of the sequencing effort would be spent on repetitive regions, which constitute up to 82% of the genomes of pine species (Wegrzyn et al., 2014). Therefore, the plant community needs cost‐efficient genotyping methods concentrating on functional regions of the genome.

Several alternatives to the WGS of the gigantic conifer genomes exist. Approaches that reduce the fraction of the genome sequenced, such as targeted sequencing (Tyrmi et al., 2020; Yeaman et al., 2016), RNA sequencing (RNA‐seq; e.g. Ojeda et al., 2019) and reduced representation sequencing (e.g. Hall et al., 2021), are in general more affordable but still have several limitations, such as additional laboratory steps (bait design, DNA fragmentation with enzymes, library preparation), allele dropouts (Andrews et al., 2016) and unequal allelic representation in RNA‐seq (e.g. Ojeda et al., 2019). They all require heavy bioinformatic processing, including read mapping, variant calling and filtering. In comparison with these methods, single‐nucleotide polymorphism (SNP) genotyping arrays are efficient and straightforward to process (Pavan et al., 2020). Genotype calls are available after relatively simple clustering analysis. They are more reproducible across studies, have fewer missing data and, importantly, require less bioinformatic preprocessing (e.g. Darrier et al., 2019). These benefits are especially valuable in species with large and repetitive genomes, such as in conifers, where the sequencing costs and bioinformatic processing of sequencing data is highest.

The SNP arrays also have limitations. For instance, they typically consist of SNPs close to or within coding regions, because data for SNP discovery are easier to obtain using RNA‐seq or exome‐targeted approaches, and thus do not represent a random set of SNPs along the genome (Neves et al., 2013). Further, coding regions are often of high interest and favored in SNP array design. As SNP arrays score preassigned SNPs with a minimum minor allele frequency (MAF) threshold, ascertainment bias affects analyses of a new sample in two ways (McTavish and Hillis, 2015). First, loci with rare alleles in the discovery population will not be scored in the new sample, causing an overestimation of loci with common alleles. Second, allele frequencies, and thus diversity, in samples genetically close to the discovery panel will be biased upwards compared with samples from a distant lineage. However, in many analyses, the ascertainment can be taken into account if the original SNP allele frequencies in the discovery panel and the SNP array design are known (Clark et al., 2005).

Among forest tree species, SNP arrays are already available for: *Juglans regia* (walnut; Marrano et al., 2019), *Picea abies* (Norway spruce; Bernhardsson et al., 2020), *Pinus taeda* (loblolly pine; Caballero et al., 2021), *Pseudotsuga menziesii* (Douglas fir; Howe et al., 2020), four European pine species (Perry et al., 2020), eight tropical pines (Jackson et al., 2022) and several eucalypt species (Silva‐Junior et al., 2015). They have been used, for instance, to build linkage maps (Silva‐Junior and Grattapaglia, 2015, Pavy et al., 2017), develop genomic selection (GS) models (Tan et al., 2017), in GWAS (Bernard et al., 2020), for candidate gene selection (Zaborowska et al., 2021) and to help conservation guidelines by informing on fine genetic structure (Silva et al., 2020).

Here, we present the Axiom PiSy50k (ThermoFisher Scientific, https://www.thermofisher.com), a new SNP genotyping array for Scots pine. We describe the SNP sources, discovery panels and selection processes used during the array design. The final array combines a set of high‐performing SNPs from a previously developed Axiom_PineGAP trans‐specific SNP array of *Pinus* (Perry et al., 2020) and a new set of curated SNPs originating from exome capture, RNA‐seq and candidate gene studies (Table 1). We provide a detailed description of SNP discovery, screening, filtering, evaluation of ascertainment bias, error rates and the metadata that we collected during the design, such as gene expression and copy‐number variation. We also explore the capability of the SNP array to discriminate populations and reconstruct pedigrees.

**Table 1:** Sources of single‐nucleotide polymorphisms (SNPs) used in the design of the PiSy50k array

Data ID	Source tissue	Ascertainment size	Sampling area	DNA/RNA	Method	Reference
a. ProCoGen haploid	M	109 haploids	Europe	DNA	Exome capture, Illumina	Tyrmi et al., 2020
b. ProCoGen diploid	N	68 diploids	Europe	DNA	Exome capture, Illumina	Kastally et al., unpubl. data
c. UOULU exomeFEB2019	NEM	2 diploids	ISS Punkaharju	DNA	Exome capture, Illumina	Kesälahti et al., unpubl. data
d. UOULU RNA‐seq	NEM	18 lineages	ISS Punkaharju	RNA	Transcriptome	Ojeda et al., 2019
e. UKCEH1^a^	N	17 diploids	Europe	RNA	SNP array Axiom_PineGAP (best set)	Perry et al., 2020
f. UKCEH2	N	17 diploids	Europe	RNA	SNP array Axiom_PineGAP; Transcriptomes of four pine species	Perry et al., 2020; Wachowiak et al., 2015
g. UOULU candidate^a^	M	12–119 haploids	Europe	DNA	Sanger sequencing, Illumina sequencing	Avia et al., 2014; Grivet et al., 2017; Kujala & Savolainen 2012; Kujala et al., 2017; Palmé et al., 2008; Pyhäjärvi et al., 2007; Vuosku et al., 2018, 2019; Wachowiak et al., 2009; Wegrzyn et al., 2008, EVOLTREE EST database (http://www.evoltree.org/index.php/e‐recources/databases/cbib)
h. LUKE candidate^a^	M	2–102 haploids	Europe	DNA	Sequence capture, Pacific Bioscience, Illumina	Kujala et al., unpubl. data; Tyrmi et al., 2020

E, embryo; M, megagametophyte; N, needle. ISS Punkaharju: Intensive Study Site Punkaharju, in south‐east Finland.

^a^ High‐priority sources, favored during the array design.

## RESULTS AND DISCUSSION

### SNP array design

**Figure 1:**
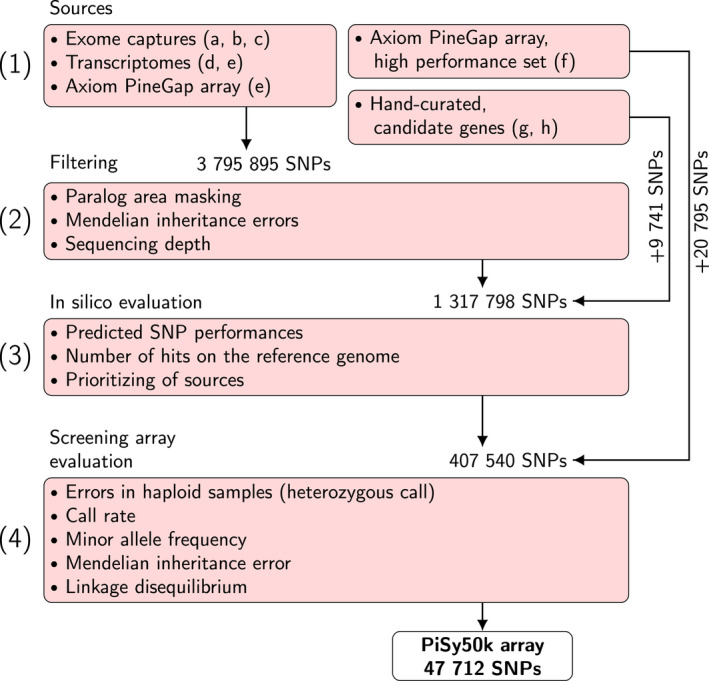
Flow chart of the PiSy50k array design. We proceeded in four steps: (1) the collection of single‐nucleotide polymorphisms (SNPs) from eight sources (a, ProCoGen haploid; b, ProCoGen diploid; c, UOULU exomeFEB2019; d, UOULU RNA‐seq; e, UKCEH2; f, UKCEH1; g, UOULU candidate; h, LUKE PacBio; Table 1); (2) filtering to remove SNPs from paralogous genomic areas, SNPs with low sequencing depth or Mendelian errors; (3) evaluation to retain the best set of 407 540 markers (screening set); and (4) filtering based on the screening array performance to select the 47 712 markers retained in the PiSy50k array.

The SNP choice and array design had four main stages: collection, filtering, *in silico* evaluation and screening array evaluation (Figure 1). We first collected SNPs from six published studies (Table 1) on Scots pine, including the transcriptome assembly (Ojeda et al., 2019), a study on genome‐wide genetic variation in Europe (Tyrmi et al., 2020), two sets from the Axiom_PineGAP SNP array (Perry et al., 2020) and two sets of candidate genes identified across multiple studies. To these six collections, we added two sets of SNPs identified in new sequencing data (Figure 1, Table 1). These eight data sets differed in sample size, sampling design, source material (RNA or DNA, tissue) and sequencing technology (Sanger sequencing, PacBio, Illumina‐seq). We filtered these initial sets, tailoring our approach to the specific characteristics of each data source. In the absence of a reference genome for Scots pine, we used the genome assembly of *Pinus taeda* (Pita 1.01; Neale et al., 2014), the best reference of a related species available when the data used in this study were first generated. As conifers have large genomes with a lot of repetitive elements, including paralogous genes (Neale et al., 2014), and as even the most recent genome assemblies are very fragmented (Pita v1.01 has a sequence length of the shortest contig at 50% of the total genome length, *N50*, of 11 216, and over 11 million contigs), the occurrence of spurious SNPs is a common problem. We relied on the patterns of genetic diversity at each locus to remove dubious markers. This was achieved by identifying genotypes from seed megagametophyte tissue (haploid), where observed heterozygosity indicates false SNPs, and in samples genotyped from needle or seed tissues (diploid) by excluding loci that deviated from the Hardy–Weinberg (HW) equilibrium and/or that had an excess of observed heterozygosity. We further filtered the data based on sequencing depth and Mendelian inheritance errors, depending on the data set. Of an initial set of 3.7 million SNPs, we submitted sequences of up to 71 nt for 1.3 million SNPs to ThermoFisher Scientific for probe design and *in silico* evaluation. This evaluation consisted in predicting the performances of the probes based on the entire set of sequences, accounting for the base composition of the sequence and the potential to map to multiple locations of the references: the reference genome (Pita v1.01) and transcriptome (Ojeda et al., 2019). From this analysis, we selected 407 540 SNPs with the best predicted performances or from candidate genes of high interest.

### Performance of the screening array

**Figure 2:**
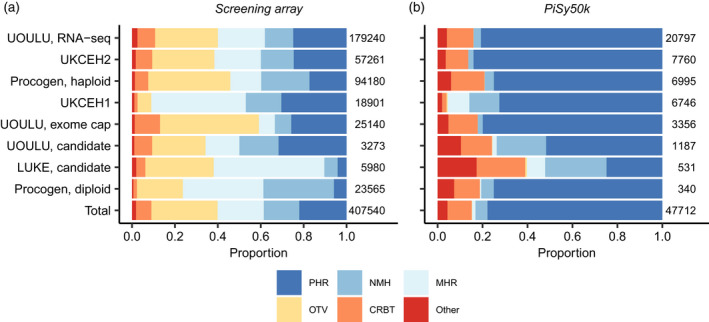
The proportions of conversion types of each marker source in (a) the screening array and (b) the PiSy50k array. Abbreviations: CRBT, call rate below threshold; MHR, mono high resolution; NMH, no minor homozygote; OTV, off‐target variant; PHR, poly high resolution. Numbers to the right of the bars indicate the total number of SNPs per marker source.

We evaluated the performance of the screening array by genotyping a natural population sample of 470 trees, six megagametophytes and four diploid embryos from full‐sib crosses, all from Finland. SNPs were assigned to six classes: poly high resolution (PHR, three well‐separated genotype clusters); no minor homozygote (NMH, two well‐separated genotype clusters, homozygous and heterozygous); mono high resolution (MHR, one homozygous genotype cluster); call rate below threshold (CRBT); off‐target variant (OTV, more than three clusters); and others. When choosing SNPs for the PiSy50k SNP array based on the screening array, we considered conversion types PHR, NMH and MHR as successful. Of 407 540 SNPs in the screening array, 245 149 (60.2%) were converted successfully and 157 325 (38.6%) were polymorphic (89 918 PHR and 67 407 NMH; Figure 2; Table S1). The success rate varied among sources from 10 to 50%, with the lowest and highest rates in the LUKE and UOULU candidate SNPs, respectively (Figure 2; Table S1). The latter set had already gone through several rounds of verification and thus the higher conversion rate was not surprising. The genotyping success rate at the sample level was high: 476 (99%) samples had a call rate above the 97% threshold in the conversion classes PHR, NMH and MHR.

**Figure 3:**
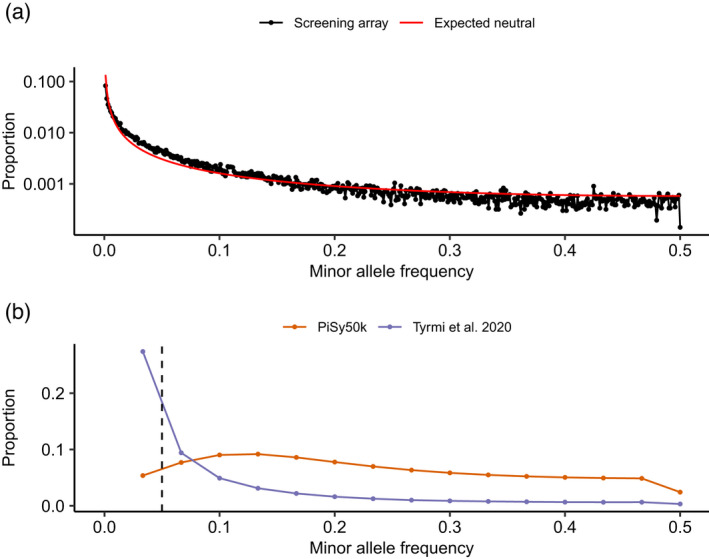
Minor allele frequency (MAF) spectra of the screening and PiSy50k arrays. (a) MAF for the screening population sample (*n* = 466) and 56 693 single‐nucleotide polymorphisms (SNPs, conversion types poly high resolution (PHR) and no minor homozygote (NMR)) without missing data in the screening array. The red line illustrates the expected neutral MAF (Tajima, 1989). Note the log scale on the *y*‐axis. (b) MAF based on the PiSy50k array, including 38 302 SNPs genotyped in 90 plus trees across three Finnish breeding populations (red line) and 42 exome captures of Scots pine trees sampled in four natural populations of Finland (Tyrmi et al., 2020). To allow comparison, we down‐sampled both distributions to 30 samples. The vertical dashed line marks the filter threshold of 0.05 used during the array design, below which SNPs were partly excluded. As expected, there is a deficiency of rare alleles in the data obtained from PiSy50k, as a result of ascertainment bias.

To assess the effects of ascertainment bias throughout the PiSy50k design, we evaluated its effects on the screening array by investigating the MAF distribution and the genetic structure in the sample. The MAF distribution of the screening array is characterized by a deficit of intermediate frequency alleles (MAF values between 0.15 and 0.50) compared with the distribution expected based on the standard neutral model (SNM) (Figure 3a). This is not surprising, as previous studies on Scots pine genetic diversity across Europe have demonstrated an overall deficit of intermediate alleles and excess of rare alleles in natural populations of this species, compared with the SNM, which could be explained by the demographic expansion of the species (Pyhäjärvi et al., 2020; Tyrmi et al., 2020, and references therein). However, the pattern of rare alleles in the screening set differs from that in earlier studies. We observed an excess of rare allele classes (MAF between 0.007 and 0.150; Figure S1), but a deficit in the extremely rare classes (MAF below 0.007; Figure S1), as expected from ascertainment bias.

In addition, ascertainment bias influenced the estimates of genetic structure among samples. Principal component (PC) analyses of the screening array genotypes of UOULU RNA‐seq and UOULU exomeFEB2019 SNPs clearly set trees included in the discovery panel separately from the rest of the samples (Figure S2). The ascertainment bias was more subtle in the other sources, even when samples from the discovery panel were genotyped (Figure S2). This difference was linked to the larger size of the other discovery panels (Table 1). The effect of ascertainment bias was particularly severe when the exact discovery panel samples or their close relatives were included (Figure S2). For most applications and data sets not related to the discovery panels, these effects on genetic structure are unlikely to be as extreme, but we recommend that users of the SNP array carefully consider sample origin when performing analyses.

Finally, from the remaining 75 629 SNPs, we excluded SNPs with heterozygous calls in megagametophyte haploid samples (but allowed one error in SNPs from three high‐priority sources, see Table 1) or with more than one Mendelian error. We also pruned SNPs in high linkage disequilibrium (LD; *r*² > 0.9), keeping the SNPs with the higher MAFs from each pair. From the remaining loci, we first retained all SNPs from high‐priority sources and favored SNPs with higher MAFs in the remaining set. SNPs in a highly outcrossing wind‐pollinated natural population of Scots pine are expected to be in HW equilibrium, hence we used deviation from HW (*P* < 0.001) to identify and filter out potentially paralogous and other error‐prone SNPs. As expected, the markers selected for the PiSy50k SNP array deviated less from the HW expectations and showed less extreme heterozygosity, compared with all screening array markers before selection (Figure S3). The final PiSy50k SNP array includes 47 712 SNPs.

### Performance of the PiSy50k SNP array

The 47 712 SNPs in the final PiSy50k SNP array were in 31 657 contigs (average of 1.5 SNPs per contig). Of the eight data sources, markers from RNA‐seq origin were the most numerous (44%; Table S2). The majority of markers have been used in previous studies and come associated with various information, depending on the source, including functional annotation, gene expression at the tissue level and allele frequency estimates in up to 20 European populations (Data S1).

**Figure 4:**
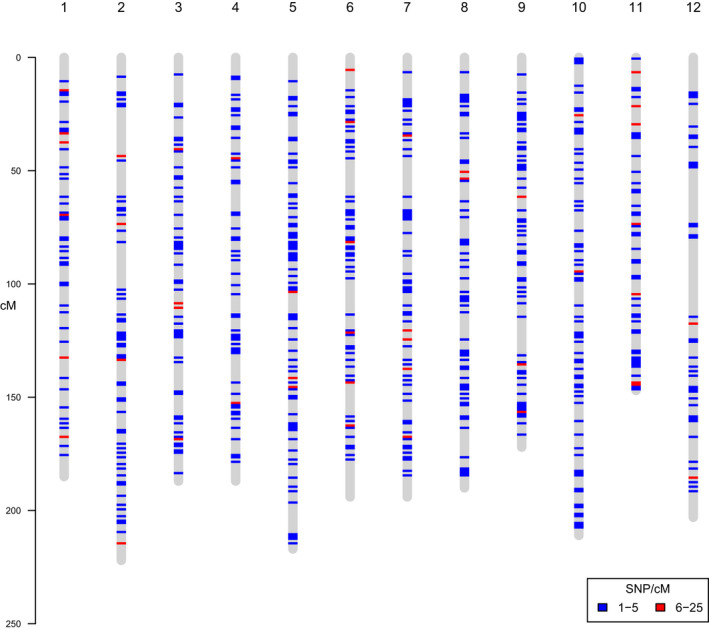
Position and density of 1619 single‐nucleotide polymorphisms (SNPs) from the PiSy50k array on the *Pinus taeda* linkage map (Westbrook et al., 2015). The vertical grey lines represent the 12 linkage groups in *P. taeda*, whereas the horizontal colored lines indicate the marker positions and densities. This plot was made with the r package chromplot 1.12.0 (Oróstica and Verdugo, 2016).

Altogether, 1619 markers derived from ProCoGen haploid (1544) and diploid sources (75) were located on one of the 4226 scaffolds mapped on the *P*. *taeda* linkage map (Westbrook et al., 2015; Figure 4; Table S3). There was an average of 134 SNPs per linkage group (LG), and they were homogeneously distributed among LGs. Even though the majority of the SNPs do not have a known position on the map yet, the quick genotyping of large numbers of progeny with the PiSy50k SNP array could be used to improve the genetic map of Scots pine and help anchor genomic reads, scaffolds and SNPs at the chromosome scale in the future.

We evaluated the performance of the PiSy50k SNP array by genotyping 2688 samples from Finland (2178, including 14 controls), Scotland (496), Australia (3) and Estonia (11). Of these, 2308 samples had call rates above 97% (85.9% of samples), the recommended threshold for Axiom SNP genotyping arrays. In total, 40 405 (84.7%) markers were successfully converted, 39 678 of which were polymorphic (Table S4).

Of the 21 control samples, three needle and six megagametophyte samples passed the 97% call rate (CR) threshold (Table S5). Of the six megagametophyte samples, one genotype was represented twice. Based on the five control samples retained (three needles and one megagametophyte pair), the error rates were relatively low (mean 0.9%). The error rate in the subset of SNPs shared with the Axiom_PineGAP suggests a similar, or slightly lower, error rate in the PiSy50k (mean 0.5% compared with 0.6% in the Axiom_PineGAP). Overall, these values are close to those obtained in other SNP arrays, e.g. 0.8% in the walnut SNP genotyping array (Marrano et al., 2019), 0.1% in Affymetrix GeneChip Human mapping 50k Array (Saunders et al., 2007) or ranging between 0.03 and 0.05% in the Axiom Apple480K SNP genotyping array (Bianco et al., 2016).

Of the 930 markers with errors among pairs (including both needle and megagametophyte controls), the majority (*n* = 916) were not shared among controls. This suggests that the error probably occurred during the genotype call for a single sample only, as opposed to the marker itself being unreliable. There are 14 markers for which errors were observed among both megagametophyte and needle controls, and these are provided in Data S2. Comparison of markers shared between the PiSy50k and Axiom_PineGAP SNP arrays (*N *= 7592) using the needle control present on both arrays also showed low error rates (mean 0.6%; Table S5) indicating cross‐array reproducibility, which allows data obtained by the two SNP arrays to be combined.

To confirm that the variants at the selected SNPs in the PiSy50k SNP array are indeed allelic (not paralog), we assessed the heterozygosity levels of the megagametophyte samples. The two megagametophyte replicates have very low heterozygosity levels (mean 0.9%) compared with the needle replicates (mean 29.3%), suggesting a low level of errors as a result of paralogy. Of the 40 405 converted markers, 38 906 were homozygous in both replicates, 1060 were ‘no call’ in at least one replicate, 165 were heterozygous in both replicates and 274 were homozygous in one replicate and heterozygous in the other. The SNPs that were heterozygous in the megagametophyte samples are indicated in Data S2.

**Figure 5:**
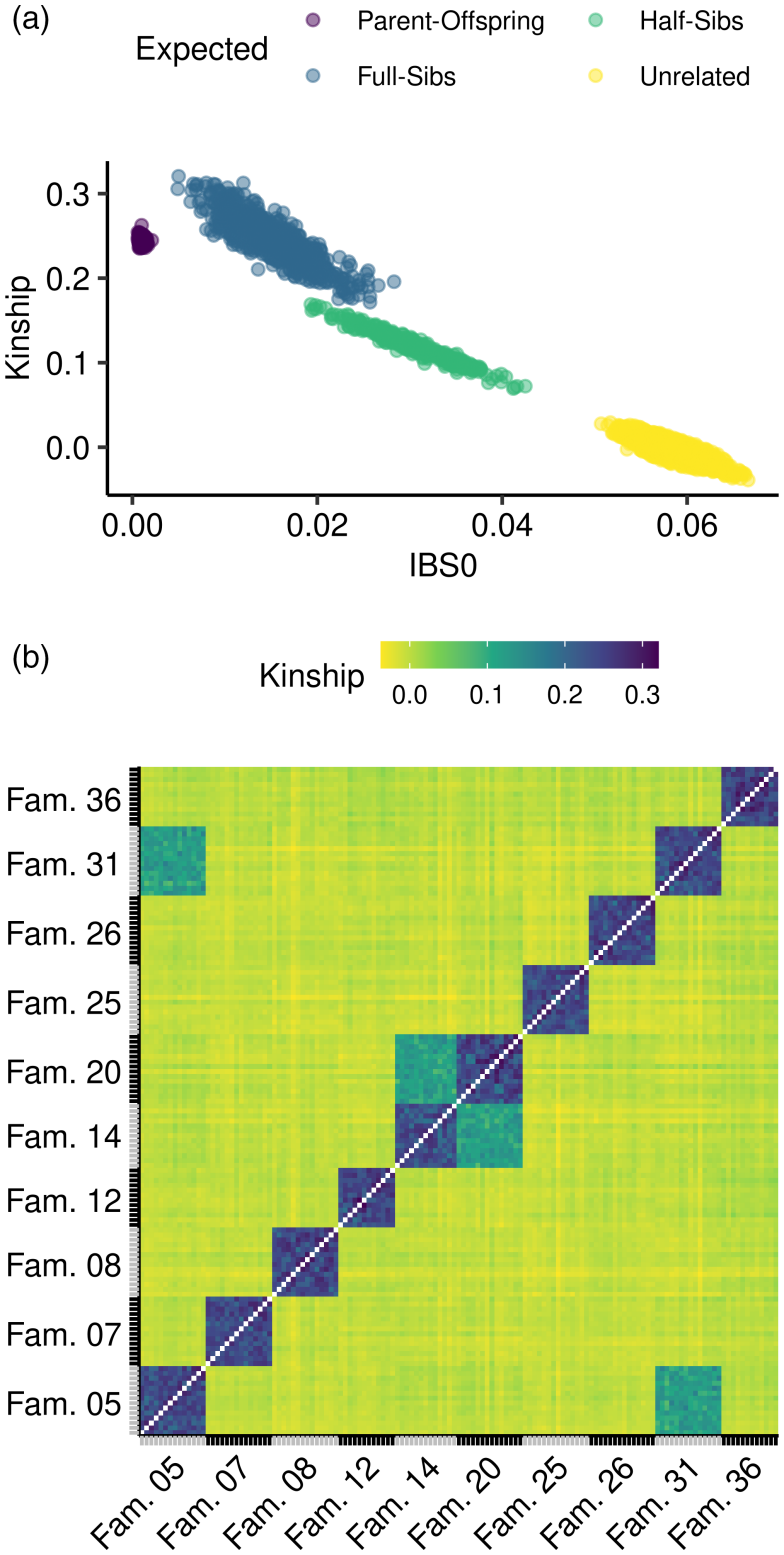
Relatedness analyses of 10 families (including 18 parents and 135 offspring) using the PiSy50k array. (a) Kinship coefficients (Manichaikul et al., 2010) and proportion of sites where individuals share no allele (IBS0) between all pairs and using 39 678 single‐nucleotide polymorphisms (SNPs) (poly high resolution (PHR) and no minor homozygote (NMR)). Expected relationships between pairs are outlined: parent–offspring in purple, full sibs in blue, half sibs in green and unrelated pairs in yellow. (b) Heat map of the kinship coefficients between all pairs of the 135 offspring.

To evaluate the potential of the PiSy50k SNP array for pedigree reconstruction and assess the proportion of Mendelian errors in the SNP array, we analyzed the pairwise relatedness of the full‐sib progeny and their parents in a subset of 135 trios across 10 families of our sample. By plotting the kinship coefficient (K; Manichaikul et al., 2010) against the proportion of sites where individuals share no allele (IBS0), we identified four distinct groups (Figure 5a): (i) known parent–offspring pairs (mean ± deviations: K = 0.245 ± 0.004, IBS0 = 0.001 ± 2e–04); (ii) full‐sibs (K = 0.246 ± 0.027, IBS0 = 0.015 ± 4e–03); (iii) half‐sibs (K = 0.120 ± 0.018, IBS0 = 0.030 ± 4e–03); and finally (iv) the remaining unrelated pairs (K = −0.002 ± 0.009; IBS0 = 0.059 ± 2e–03). We separated parent–offspring pairs from full‐sibs, which have expected K values close to 0.250, using the IBS0 statistic (equal or close to 0 between a parent and an offspring, but with higher values between siblings; Manichaikul et al., 2010). Within each family, the K estimates were around the expected value of 0.250, whereas between families K was close to 0, except for progeny pairs between families 5 and 31 and families 14 and 20, which shared a common parent and had a K estimate of around 0.125, as expected for half‐sibs (Figure 5). The pedigree relationships identified with PiSy50k matched those expected from the crossing design, demonstrating the power of the SNP array to resolve relatedness structure and reconstruct pedigrees, a critical feature for a multitude of applications in tree breeding and genetics: GWAS, GS, breeding program management and seed production.

To further assess the error rate in the PiSy50k data, we evaluated the number of Mendelian errors (MEs) within each family. We examined all 40 405 SNPs in 135 trios and identified 16 040 errors across 5837 loci (mean error rate per locus = 0.3%; Figure S4). More than 98% of all SNPs had an ME below 5%. Across families, we identified an average of 1604 errors per family, with the majority in different SNPs across families (4277 SNPs with an error only in a single family and 1110 in at least two; Figure S4). These values are in line with the MEs measured in other SNP arrays (Bernhardsson et al., 2020; Silva‐Junior et al., 2015).

### Genetic diversity

**Figure 6:**
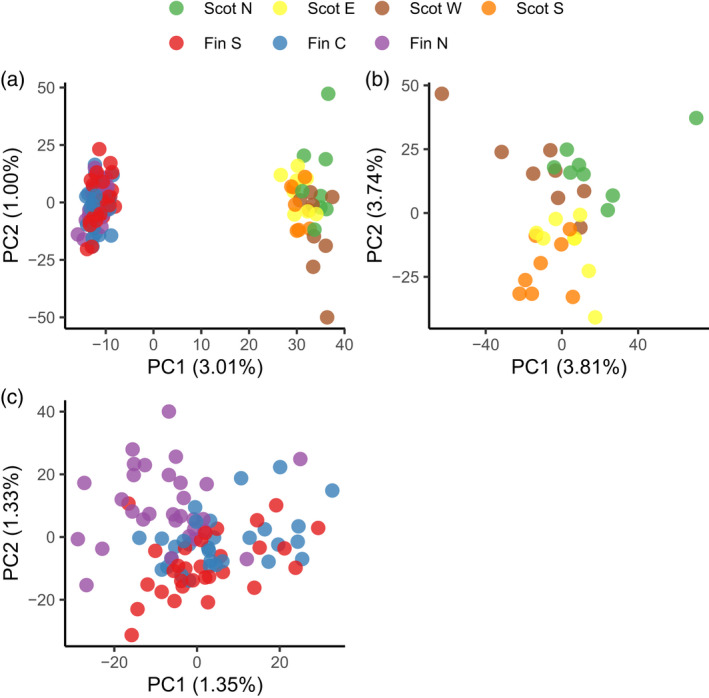
Principal component analysis (PCA) using 39 678 polymorphic single‐nucleotide polymorphisms (SNPs) from the PiSy50k array genotyped in 122 trees from seven areas in Finland (90) and Scotland (32). PCA including (a) all 122 samples from Finland and Scotland, (b) 32 samples collected across 21 localities grouped into four geographical areas of Scotland or (c) 90 samples from southern, central and northern Finland (30 samples each). Scot N, E, W and S: northern, eastern, western and southern Scotland. Fin S, C and N: southern, central and northern Finland.

To explore the power of genotypes from the PiSy50k SNP array to discriminate trees from different geographic origins, we ran a principal component analysis (PCA) using a subset of 122 samples from different localities in Scotland and Finland (Figure 6). The first two PCs separated two main groups, consistent with the two countries of origin. We then ran PCAs using only samples within each country. Although no distinct groups appeared in those analyses, some differentiation was found between samples from different geographic origins in Scotland (Figure 6b) – a level of geographic resolution not previously possible. In the Finnish subset, variation was more homogeneous with less geographic structure (Figure 6c), although samples from northern origins were located slightly apart from samples from southern and central origins.

To assess the effects of ascertainment bias on the MAF distribution in the PiSy50k SNP array, we compared the frequency distributions obtained from the array with a previously published exome capture data set (Tyrmi et al., 2020) (Figure 3b). We observed a similar but stronger effect of ascertainment on the MAF estimated with the PiSy50k SNP array genotyping results than with the screening array results. Indeed, in the PiSy50k results, the distribution reaches a maximum at frequency 0.13, with decreasing frequencies of lower MAF values, as opposed to the screening array where the peak is at the lowest allele frequency class. This could be explained by the more stringent filtering of SNPs with low allele frequencies when selecting markers for the final PiSy50k set, whereas there was no intentional allele frequency filtering from the source data to the screening set. In addition, the discovery process naturally has an inherent filter for allele frequency, which is the sample size of the discovery panel.

In summary, PiSy50k is a novel SNP genotyping array for Scots pine, an economically important and widely distributed conifer. The low error rates and high reproducibility obtained in control samples, including comparisons with genotyping results from the Axiom_PineGAP SNP array designed for four European pine species, including Scots pine (Perry et al., 2020), indicate that data produced with PiSy50k are reliable and will allow comparison across studies. Moreover, the metadata provided connects the genotyping data to functional properties via annotations and tissue‐specific expression patterns. This new SNP array greatly improves the genotyping capacity for Scots pine, which will facilitate future breeding and evolutionary research, e.g. to perform genomic selection, pedigree construction, GWAS and genetic mapping. Genetic mapping and association analyses will help to disentangle the genetic architecture of traits and, together with gene expression data, understand the molecular basis of, for example, adaptive phenotypic traits that are striking in forest trees (Alberto et al., 2013).

## EXPERIMENTAL PROCEDURES

### Selection of SNPs for initial screening

#### ProCoGen haploid and diploid sets

The ProCoGen haploid and diploid sets were generated with two exome‐capture experiments, both based on the same bait set used by Tyrmi et al. (2020). A total of 177 trees collected across Europe, from Spain to northern Finland, were genotyped using DNA extracted from megagametophyte tissue (haploid set, 109 samples, 12 populations) or needles (diploid set, 68 samples, 8 populations). Bait design, DNA extraction, library preparation and sequencing steps followed the procedure described by Tyrmi et al. (2020). We processed the sequences generated to identify SNPs following the same method described by Tyrmi et al. (2020) for the haploid set, but applied a few adjustments for the diploid set: we used bwa (Li, 2013) for mapping reads and used samtools 0.9 (command *mpileup*, with default parameters; Li et al., 2009) for variant calling. To filter potential paralogs, we removed loci with heterozygous calls in the haploid set or loci significantly departing from HW equilibrium in the diploid set (plink 1.90b5.2, using command ‐‐hardy, excluding SNPs with *P* < 0.05; Chang et al., 2015). During this procedure, we excluded one haploid sample with an exceptionally high proportion of heterozygous calls. Finally, we excluded all SNPs within 50 bp distance of these markers. We retained 248 591 and 32 649 SNPs in the haploid and diploid sets, respectively.

#### UOULU exomeFEB2019

We used 95 504 SNPs identified in exome capture of a family originating from Punkaharju Intensive Study Site (ISS), in south‐east Finland: a cross between maternal tree 463 and paternal tree 485 (Kesälahti et al., unpubl. data). The material sampled consisted of needles of both parental trees, one megagametophyte of the paternal tree, two megagametophytes of the maternal tree from open‐pollinated seeds and, from two seeds of the cross progeny, two embryos and a megagametophyte. We excluded positions with depths of <4 per genotype. We removed twenty‐five base pairs, both upstream and downstream, from each heterozygous site found in haploid megagametophyte as potential areas with paralog or mapping issues.

#### UOULU RNA‐seq

The UOULU RNA‐seq set refers to markers derived from RNA‐seq data (Ojeda et al., 2019) originating from five tissues (needle, phloem, vegetative bud, embryo and megagametophyte) of six unrelated individuals of Scots pine (but 18 haploid genomes when accounting for diploidy and paternal contribution in embryos) collected from Punkaharju ISS. We considered 1 349 291 SNPs obtained by mapping RNA‐seq reads to the Scots pine reference transcriptome (https://www.ncbi.nlm.nih.gov/nuccore/GILO00000000.1). From this initial set, we first excluded markers identified in contigs associated with potential contaminants (fungi or microbes; Cervantes et al., 2021; Ojeda et al., 2019; https://figshare.com/articles/dataset/Pinus_sylvestris_assembly_Trinity_guided_gene_level_information/13109492/1). Second, we removed heterozygous SNPs in haploid samples. Finally, we compared the genotypes called in megagametophyte, embryo and diploid tissues collected from the same tree to identify and exclude loci with Mendelian errors. In total, we retained 736 827 SNPs.

For the UOULU RNA‐seq set, we provide information about the predicted multicopy status, orthologous genes identified in *P*. *taeda* (Zimin et al., 2014) and *Pinus lambertiana* (Stevens et al., 2016) based on BLASTN results (see details in Ojeda et al., 2019), and expression levels and tissue specificity in five tissues (Cervantes et al., 2021). This information is available in Data S1.

#### UOULU candidate

The UOULU candidate set contains SNPs reported in multiple publications and genetic databases on various candidate genes of Scots pine. This set includes the SNP markers used by Kujala et al. (2017), and additional SNPs from phenology‐related genes (Kujala and Savolainen, 2012; Palmé et al., 2008; Pyhäjärvi et al., 2007, Wachowiak et al., 2009), stress and phenology‐related genes (Avia et al., 2014), polyamine genes (Vuosku et al., 2018, 2019), genes from comparative resequencing projects (Wegrzyn et al., 2008; Grivet et al., 2017) and markers identified in sequences from the EVOLTREE expressed sequence tag (EST) database (http://www.evoltree.org/index.php/e‐recources/databases/cbib; taken from two *P. sylvestris* cDNA libraries, from needles, including 9059 and 18095 sequences). Additionally, for a subset of those markers, we collected allele frequency estimates from two genotyping assay experiments on 426 Scots pine trees (Avia et al., unpubl. data). These SNPs, referred to as UOULU candidate VIP in the metadata, were given higher priority during the SNP array manufacture, in both the screening and PiSy50k SNP arrays, by increasing their probe‐set counts and, in this way, improving their call rates during the genotyping.

#### LUKE candidate

The LUKE candidate set comprises SNPs extracted from candidate genes related to phenology (e.g. Bouché et al., 2016) and genes of the primary and secondary metabolism pathways active during heartwood formation (Lim et al., 2016). DNA libraries targeting these candidate genes were produced from one individual of Southern Finnish origin and sequenced using a PacBio sequencer (https://www.pacb.com) (Kujala et al., unpubl. data). We used the long PacBio sequences as a reference to map short reads from exome captures of megagametophyte samples of Scots pine collected across Europe (Tyrmi et al., 2020; excluding samples from Baza, Spain) with bwa‐mem (Li, 2013). As a preliminary variant calling based on this initial mapping resulted in a large number of errors (heterozygous calls in haploid samples), we isolated short reads mapping to individual PacBio contigs and reassembled them with mira (Chevreux, 2007) for each individual. We then aligned the resulting individual re‐assemblies with each other using cap3 (Huang and Madan, 1999), and called variants using bcftools (with commands *mpileup* and *call*). In addition, some SNPs were identified and included solely as being polymorphic within the reference individual.

#### UKCEH sets 1 and 2

We used SNPs collected during the Axiom_PineGAP (ThermoFisher Scientific) array design (Perry et al., 2020) and from the comparative transcriptomics of four pine species (*P*. *sylvestris*, *Pinus mugo*, *Pinus uncinata* and *Pinus uliginosa*) published by Wachowiak et al. (2015). Briefly, we identified 196 636 polymorphic positions from transcriptomes, candidate gene sequences and markers from previous population genetic studies on the four pine species mentioned above. From these, we retained two distinct sets: (i) UKCEH1, comprising 20 795 successfully converted SNPs from the Axiom_PineGAP array; and (ii) UKCEH2, a set of 175 841 SNPs, including 29 034 SNPs from the Axiom_PineGAP array that were not successfully converted, 31 897 SNPs that passed the initial filtering during the design but were not included in the final array and 114 910 SNPs identified by Wachowiak et al. (2015), which were polymorphic in Scots pine but not included in the Axiom_PineGAP array design.

### SNP scoring for inclusion in the screening array

For each retained site, we built 71‐mer probes by extracting up to 35 bp up‐ and downstream from the source references. We submitted 1 317 798 probes to the Microarray Research Services Laboratory (ThermoFisher Scientific) for scoring (Table S1). During this step, probe scores were downgraded if they contained polymorphic sites within 35 bp of the focal marker (interfering polymorphism), they were mapped to highly repetitive regions of the genome (using TrinityCD‐HIT.fasta.gz and Pita v1.01 as references for RNA‐ and DNA‐based probes, respectively; https://treegenesdb.org/FTP/Genomes/Pita/v1.01/genome/Pita.1_01.fa.gz) or were highly similar to other probes. Each marker was given a classification: ‘recommended’, ‘neutral’, ‘not recommended’ or ‘not possible’.

Based on the evaluation from ThermoFisher Scientific and the available metadata on each data source, we established the following priority groups (in order of priority): (i) the 20 795 high‐quality SNPs from the Axiom_PineGAP array; (ii) all recommended or neutral markers identified by ThermoFisher Scientific; (iii) UOULU candidate markers; (iv) LUKE candidate markers; (v) markers from the ‘not recommended’ set in the ProCoGen haploid set, including SNPs of high interest identified by Tyrmi et al. 2020); (vi) SNPs with less than 50% of missing data in the discovery panel from the ‘not recommended’ set in the ProCoGen sets; and finally (vii) we relaxed the filtering criterion used by ThermoFisher Scientific and selected the best markers in the remaining set. More specifically, we relaxed the wobble count filter threshold (number of polymorphic sites on the same 71‐mer) from <4 to <6, based on the assumption that a high proportion of the variable sites are associated with rare alleles, and thus interfering polymorphism should have a lower impact on probe performance in the case of Scots pine. During the manufacture of the screening array, out of the 428 516 SNPs retained, a total of 407 540 markers were fitted on the array.

### Screening set genotyping

The screening set of 407 540 SNPs was used to confirm the normal segregation of polymorphism in a larger sample from a natural population, to identify potential deviations from HW equilibrium, indications of paralog mapping, such as heterozygote sites in haploid samples, deviations from Mendelian segregation and the identification of loci in strong LD with each other. To this end, we used the screening array to genotype 480 samples of Scots pine from the Punkaharju ISS population, including: 470 diploid needle samples from adult trees, six haploid megagametophytes and four diploid embryos. Two families, ‘463 × 485’ and ‘320 × 251’, with two parents and two offspring (embryos) from each, were used to estimate the Mendelian error rate.

DNA was extracted from dry needles and fresh megagametophytes using the E.Z.N.A.^®^ SP Plant DNA Kit (Omega Bio‐tek, https://www.omegabiotek.com). Genotyping and array manufacturing for the screening set was performed by ThermoFisher Scientific. Genotype calling was performed by ThermoFisher Scientific (Applied Biosystems^TM^ Axiom^TM^ Genotyping Services) following the Axiom Best Practices Workflow (Axiom Genotyping Solution Data Analysis Guide). In short, genotype clusters were defined using samples with a quality control call rate (QCCR) ≥ 0.97 and dish quality control rate (dQC) ≥ 0.82. The markers were classified into five conversion categories: PolyHighResolution (PHR), NoMinorHom (NMH), MonoHighResolution (MHR), CallRateBelowThreshold (CRBT), Off‐Target Variant (OTV) and other. We visually inspected a random set of 200 genotype clusters from each conversion category to check that the genotype clusters were clearly separated. We retained markers only from classes PHR and NMH with CR ≥ 0.97 in the subsequent analyses of the screening array and for inclusion on the PiSy50k SNP array.

During the design of both screening and PiSy50k SNP arrays, identical SNPs discovered independently across different sources were identified and merged. To keep track of as much information as possible for those markers, we recorded their common presence and IDs in different sources but eventually assigned a single authoritative origin.

### Selection of markers for the PiSy50k SNP array

For the PiSy50k SNP array, we filtered the markers based on their performance on the screening array, prioritizing markers in candidate genes of interest or markers that performed well in the Axiom_PineGAP array (Perry et al., 2020; additional information provided in Appendix S1). These markers were within the Axiom Best Practices Workflow default quality thresholds (see above). For each marker with conversion type PHR or NMH, we estimated MAF and tested departure from HW equilibrium (exact test) for 466 individuals, excluding the haploid megagametophyte samples, the offspring samples and four samples with QCCR < 0.97 using plink 1.9 (Purcell et al., 2007). We estimated the number of Mendelian errors in plink using the family data.

We excluded markers deviating from HW equilibrium (*P* < 0.001) and markers with more than one Mendelian error. Markers from the candidate gene sources (LUKE candidate and UOULU candidate) were selected using a lenient inclusion threshold of MAF ≥ 0.01 and marker CR > 0.90, which also included markers from the ThermoFisher Scientific conversion type ‘call rate below threshold’. We filtered SNPs from the Axiom_PineGAP array first to include markers with MAF ≥ 0.05. To increase the number of well‐performing markers, we also included markers with MAF ≥ 0.05 in previously genotyped European samples (Perry et al., 2020).

To avoid markers in paralogous genomic regions, we excluded markers with heterozygous call in the haploid megagametophyte samples except in three high‐priority sources (UKCEH1, LUKE candidate and UOULU candidate), for which we allowed at most one, erroneous, heterozygous call per marker. We further granted 358 markers of high interest from sources UOULU candidate (335) and UOULU RNA‐seq (23) a higher probe‐set count in the SNP array to increase their call rate. Finally, to remove the excess from the retained set, we excluded markers from the low‐priority sources with lowest MAFs (MAF ≥ 0.08 after filtering). The final number of markers for PiSy50k was 47 712 (Figure 1). The distribution of the markers by source is shown in Table S2.

To inspect how SNP selection for the PiSy50k SNP array affected HW deviation compared with the screening array on average, we plotted the observed *P* values from the exact HW tests against the expected *P* values based on the null distribution in a cumulative quantile–quantile (Q–Q) plot before and after SNP selection. We compared the observed *P* values of 10 000 random loci against 100 samples drawn from the null distribution using the hardyweinberg package (Graffelman, 2015) in r 3.6.3 (The R Project for Statistical Computing). We also illustrated the distribution of genotypes with respect to HW expectations in ternary plots showing genotypes before and after the PiSy50k SNP choice.

To assess the effects of ascertainment bias on the screening array, we ran two analyses. First, we plotted the MAF distribution for loci with conversion types PHR or NMH (no. loci without missing data = 56 693; no. individuals = 466) against the expected MAF assuming a standard neutral model (Tajima, 1989). Second, we looked at the effects of ascertainment bias on the inference of genetic structure by conducting PCAs using the r package pcadapt (Privé et al., 2020). We performed PCAs using SNPs separately from each source and retained the results from two sets where we observed the strongest effects of ascertainment bias, from sources UOULU RNA‐seq and UOULU exomeFEB2019, and one in which the effects were minimal, the ProCoGen haploid sources. To further illustrate the root cause of the observed biases, we performed those PCAs with and without the individuals present in the original discovery panel and driving the patterns observed.

### Linkage map position of PiSy50k markers

To assess whether markers from the PiSy50k SNP array are homogeneously distributed across all chromosomes, we positioned them on a genetic map produced for *P*. *taeda* by Westbrook *et al*. (2015), comprising 12 linkage groups (LGs), to which contigs from *P*. *taeda* reference genome Pita v1.01 have been mapped. We included all PiSy50k SNPs previously mapped to one of the contigs or scaffolds from the same reference genome (data sources ProCoGen haploid and diploid). When a given SNP was outside the aligned segment of the reference contig, we used the closest position effectively aligned on the genetic map from the same contig as a reference point to infer the position of the focal SNP on the map, assuming the physical distances covered by single contigs from the Pita v1.01 reference genome to be negligible compared with the size of each individual LG.

### PiSy50k SNP array genotyping

We tested the PiSy50k SNP array performance by genotyping 2688 samples (across seven plates). The 2688 samples consisted of 317 Finnish plus trees, 1847 full‐sib offspring from the Finnish breeding population, 489 Scottish samples, three Australia samples, 11 Estonian samples and 21 controls. The needle control was a single tree from Scotland, UK, and was included on each genotyping plate; this sample had also been genotyped on the Axiom_PineGAP array. In addition, seven haploid megagametophyte samples were genotyped twice, such that each sample was genotyped on two different random plates. Other samples were randomized over the plates such that the different geographic locations and sample categories (plus trees and offspring) were spread on all plates to avoid plate effects that could bias the genotyping results of a specific sample category.

The SNP arrays were manufactured by ThermoFisher Scientific and genotyping was conducted by University of Bristol Genomics Facility (Bristol, UK). Needle samples (*n* = 2 674, including seven controls) were dried and stored in bags with silica gel. For megagametophyte samples (seven control samples included twice each), germination was initiated by placing the seeds on moist filter paper inside a Petri dish for 24 h at room temperature (~21°C). Seeds were then dissected under a microscope to separate megagametophyte from the embryo tissue. The DNA from Finnish and Estonian samples was extracted using E.Z.N.A.^®^ SP Plant DNA Kit (Omega Bio‐tek). The DNA of Scottish needles was extracted using a Qiagen DNeasy Plant kit (https://www.qiagen.com) and checked visually on a 1% agarose gel. DNA was quantified with a Qubit spectrophotometer (ThermoFisher Scientific).

We performed the genotype call using axiom analysis suite 5.1.1.1, following the Axiom Best Practices Workflow with default parameters concordantly with the screening array genotype calling, except for the plate QC threshold for average call rate for passing samples, which we set to 0.97. We visually inspected 50 genotype clusters from each conversion class and retained all markers in categories PHR and NMH for analyses.

### Evaluation of the PiSy50k SNP array performance

#### Error rate and heterozygosity in haploid samples

We genotyped 21 control samples to estimate error rates for each SNP array: one needle and two megagametophyte controls per plate, with replicate megagametophyte pairs arranged over sequential plates. We estimated the error rates as the proportion of calls that did not match among pairs of controls across plates (excluding calls where one or both were missing). We also measured the heterozygosity in megagametophyte samples to assess probe specificity and identify putative paralogous markers in the PiSy50k SNP array.

#### Pedigree inference and Mendelian error rate

We used a subset of 153 samples from 10 crosses, including 18 parents and their 135 offspring, to estimate the coefficients of kinship (K) and the proportion of sites where individuals share no allele (IBS0) between all pairs using converted SNPs (40 405) with king 2.2.5 (options ‐‐related ‐‐degree 2) (Manichaikul et al., 2010). We estimated the Mendelian error rate within each family independently using plink 1.90b5.2 (option ‐‐mendel).

#### Population clustering and ascertainment bias

To evaluate the power of PiSy50k in discriminating samples from different origins, we used a subset of 122 plus tree samples: 32 samples from Scotland, grouped in four geographic areas, and 30 samples each from southern, central and northern Finland. We assessed the genetic structure by performing three PCAs: using all 122 samples and using the 90 Finnish samples or the 32 Scottish samples separately. We used the function *prcomp* (R Core Team, 2018, with scaling and centering options enabled) after replacing missing data for a given genotype by the allele frequency of the locus. Finally, to assess the effect of ascertainment bias on the MAFs generated with PiSy50k, we compared the MAF distribution of the Finnish subset of 90 plus trees to that obtained using exome capture data of Scots pine trees published in Tyrmi et al. (2020). From the published .vcf file, we extracted the data of 42 megagametophyte samples from four Finnish populations (Inari, Kälviä, Kolari and Punkaharju). We then replaced genotypes with depths of <5 with missing data and only retained loci with a minimum call rate of 50%. Finally, to obtain comparable MAF distributions, we down‐sampled both distributions to a sample size of 30.

## AUTHOR CONTRIBUTIONS

Design of the study: AKN, AP, CK, KK, MH, OS, StC, STK and TP. Field and laboratory work: AKN, AP, SaC, STK, TAK, TP, RK and StC. Computational analyses: AKN, AP, CK, DIO, JST, KA, STK, TMM, TP and WW. Initial draft of the article: AKN, CK and TP. Final article: all authors.

## CONFLICT OF INTEREST

The authors declare that they have no conflicts of interest associated with this work.

## Supporting information


**Figure S1.** Minor allele frequencies for the Punkaharju Intensive Study Site (south‐east Finland) population (*n* = 466) and 56 693 SNPs without missing data in the screening array.
**Figure S2.** Principal component analysis on the screening array data, illustrating ascertainment bias on the observed genetic structure.
**Figure S3.** Hardy–Weinberg equilibrium (HW) test results for the screening array data before filtering and for the selected set for PiSy50k.
**Figure S4.** Mendelian errors (MEs) of the PiSy50k identified in 40 405 SNPs genotyped in 135 trios (10 crosses).
**Table S1.** Conversion type for markers from each data set in the screening array based on individuals with call rates of 97% or above.
**Table S2.** Number and proportions of markers from each source at different steps of the PiSy50k SNP array design.
**Table S3.** Distribution of PiSy50k markers on *Pinus taeda* linkage groups (Westbrook et al., 2015).
**Table S4.** Conversion type for markers from each data set in the PiSy50k SNP array based on individuals with call rates of 97% or above.
**Table S5.** Evaluation of the PiSy50k SNP array for control samples with call rates of 97% or above.
**Appendix S1.** Additional steps/details in selecting markers from screening array to PiSy50k SNP array.

**Data S1.** The metadata for markers included on the PiSy50k SNP array.
**Data S2.** Shared errors across controls identified in the error evaluation of the PiSy50k, see the main text.
**Data S3.** Genotypes of 268 samples used in this study including 10 progenies and trees from Finland and Scotland.

## Data Availability

All relevant data can be found within the article and its supporting materials. Metadata of all SNPs are included in the file Data S1. Genotype data for the 268 samples used in this article are available in .vcf format in the file Data S3, including 10 progenies (135 offspring and their parents) and trees from Finland and Scotland (122 samples).
