# Supplementary material for: Taming the massive genome of Scots pine with PiSy50k, a new genotyping array for conifer research
Source: Plant J. 2022 Jan 16;109(5):1337–50. doi: 10.1111/tpj.15628 (PMC9303803; doi:10.1111/tpj.15628)
Supplement: Supplementary file 1 — Figure S1. Minor allele frequencies for the Punkaharju Intensive Study Site (south‐east Finland) population (n = 466) and 56 693 SNPs without missing data in the screening array. Figure S2. Principal component analysis on the screening array data, illustrating ascertainment bias on the observed genetic structure. Figure S3. Hardy–Weinberg equilibrium (HW) test results for the screening array data before filtering and for the selected set for PiSy50k. Figure S4. Mendelian errors (MEs) of the PiSy50k identified in 40 405 SNPs genotyped in 135 trios (10 crosses). Table S1. Conversion type for markers from each data set in the screening array based on individuals with call rates of 97% or above. Table S2. Number and proportions of markers from each source at different steps of the PiSy50k SNP array design. Table S3. Distribution of PiSy50k markers on Pinus taeda linkage groups (Westbrook et al., 2015). Table S4. Conversion type for markers from each data set in the PiSy50k SNP array based on individuals with call rates of 97% or above. Table S5. Evaluation of the PiSy50k SNP array for control samples with call rates of 97% or above. Appendix S1. Additional steps/details in selecting markers from screening array to PiSy50k SNP array. [file TPJ-109-1337-s002.pdf]

Supporting information

Taming the massive genome of Scots pine with PiSy50k, a new genotyping array for conifer research

Chedly Kastally[a]*, Alina K. Niskanen[a]*, Annika Perry[b], Sonja T Kujala[c], Komlan Avia[d], Sandra Cervantes[a], Matti Haapanen[e], Robert Kesälahti[a], Timo A Kumpula[a], Tiina M Mattila[a,f], Dario I. Ojeda[a,g], Jaakko S. Tyrmi[a], Witold Wachowiak[h], Stephen Cavers[b], Katri Kärkkäinen[c], Outi Savolainen[a], Tanja Pyhäjärvi[a,i]**

[a]Department of Ecology and Genetics, University of Oulu, 90014 University of Oulu, Finland

[b]UK Centre for Ecology & Hydrology, Bush Estate, Penicuik, Midlothian, UK, EH26 0QB, UK.

[c]Natural Resources Institute Finland (Luke), Paavo Havaksen tie 3, 90570 Oulu, Finland

[d]Université de Strasbourg, INRAE, SVQV UMR-A 1131, F-68000, Colmar, France

[e]Natural Resources Institute Finland (Luke), Latokartanonkaari 9, FI-00790 Helsinki, Finland

[f]Department of Organismal Biology, EBC, Uppsala University, Uppsala, Sweden

[g]Norwegian Institute of Bioeconomy Research, Ås, Norway

[h]Institute of Environmental Biology, Faculty of Biology, Adam Mickiewicz University in Poznań, Uniwersytetu Poznańskiego 6, 61–614 Poznań, Poland

[i]Department of Forest Sciences, University of Helsinki, 00014 University of Helsinki, Finland

* Contributed equally

** Corresponding author: Tanja Pyhäjärvi, Department of Forest Sciences, University of Helsinki, FIN-00014 University of Helsinki, Finland, tanja.pyhajarvi@helsinki.fi

This document includes:

- Figures S1 to S4
- Tables S1 to S5
- Appendix S1: "Additional steps/details in selecting markers from screening array to PiSy50k array"

Other supporting materials for this manuscript include the following:

- Data S1: the metadata for markers included on the PiSy50k array.
- Data S2: shared errors across controls identified during the error evaluation of the PiSy50k.
- Data S3: genotypes of 268 samples used in this study, including 135 offspring (and their parents) from 10 progenies and 122 trees from Finland (90) and Scotland (32)

1 Supporting figures

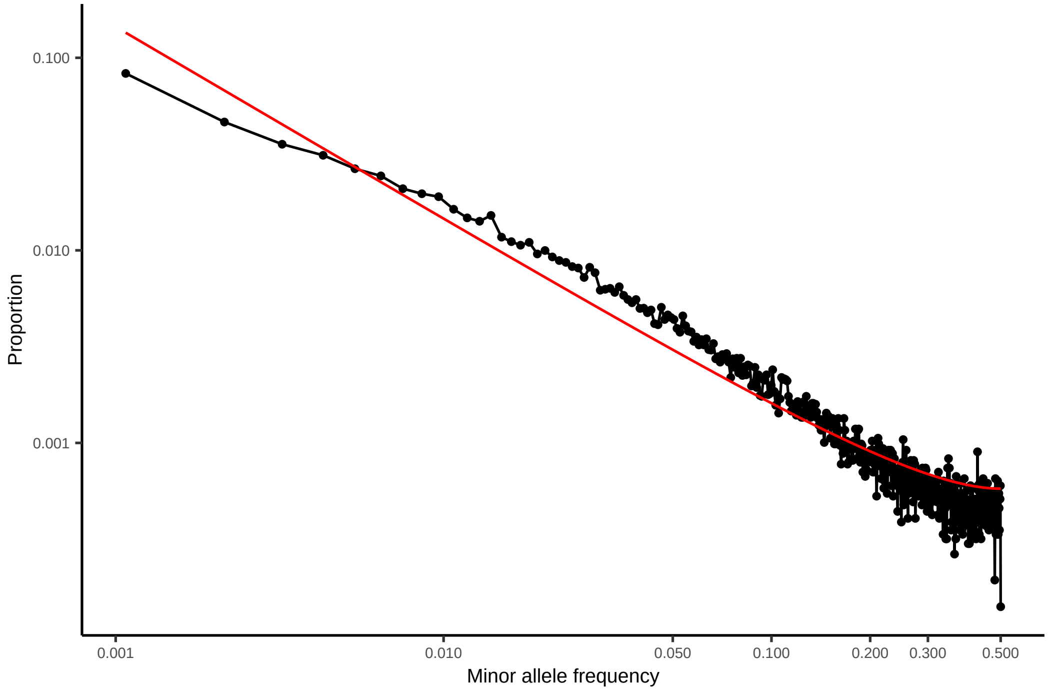

Figure S1. Minor allele frequencies for the Intensive Study Site Punkaharju (southeast Finland) population (N=466) and 56 693 SNPs without missing data in the screening array. The red line illustrates the expected neutral MAF (Tajima, 1989). Note that this figure is identical to Figure 3 but is represented with a logarithmic scale on both the x- and y-axes.

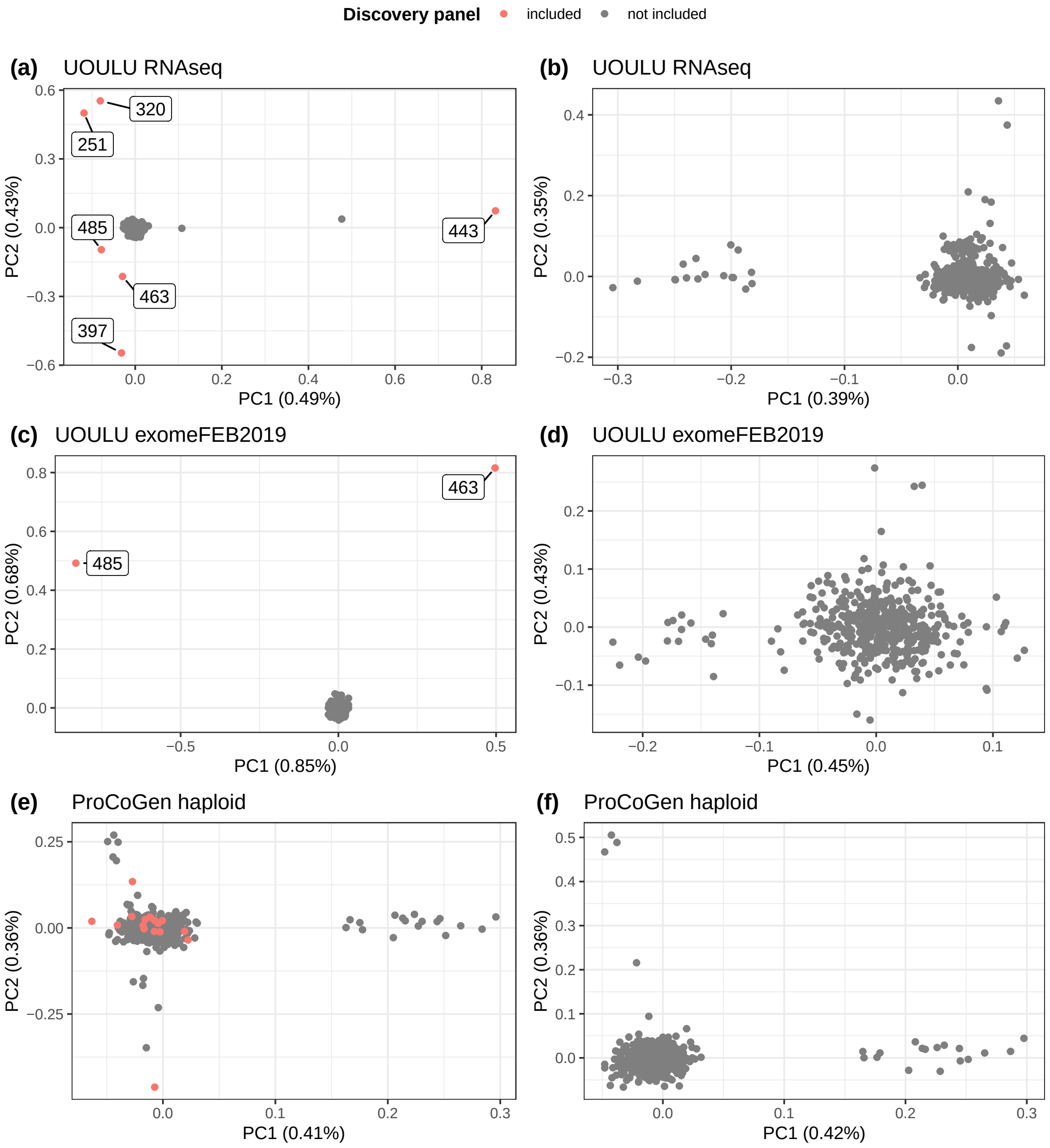

Figure S2. Principal component analysis on the screening array data illustrating the ascertainment bias on the observed genetic structure. (a, c, e) Analysis including samples used in SNP discovery panels of each SNP source, discovery individuals are highlighted and labelled, except in e) for clarity. (b, d, f) Analysis excluding samples used in SNP discovery. SNP sources: (a, b) UOULU RNA-seq (48 357 SNPs), (c, d) UOULU exome-FEB2019 (6 137 SNPs) and (e, f) ProCoGen haploid (23 204 SNPs).

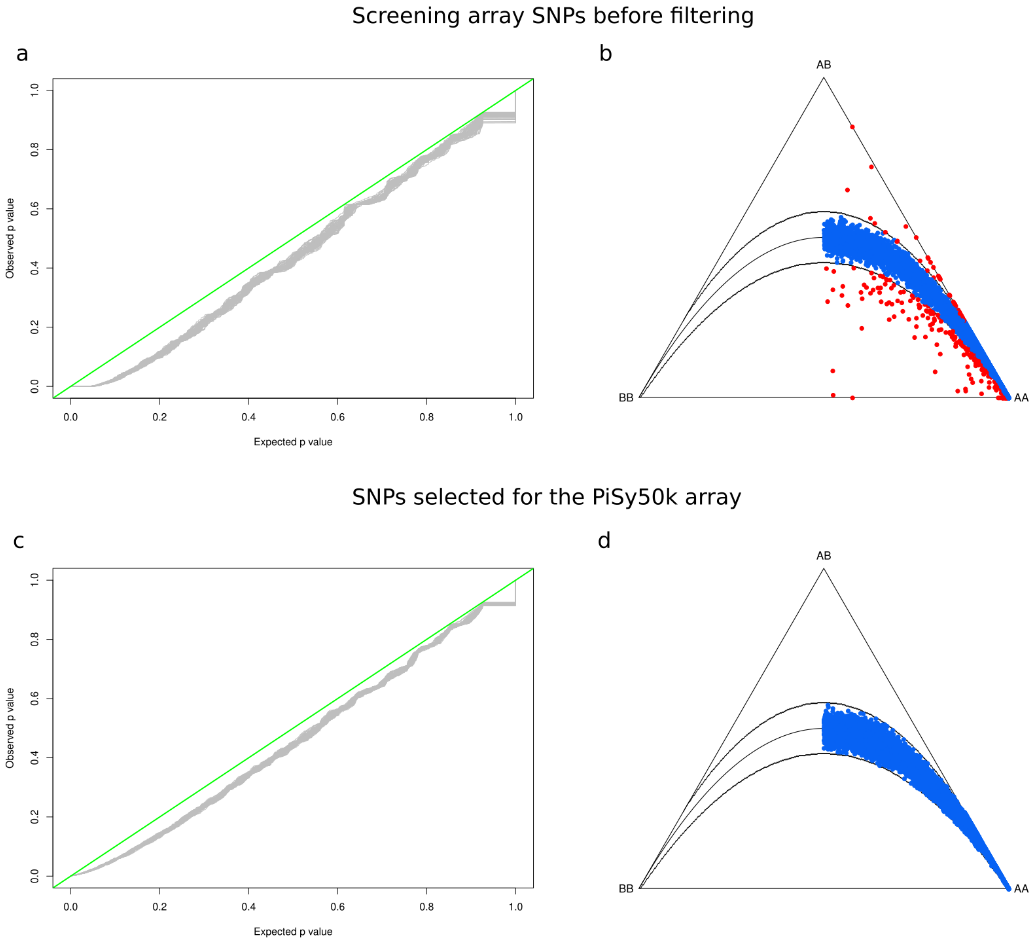

Figure S3. Hardy-Weinberg equilibrium (HW) test results for the screening array data before filtering (a,b) and for the selected set for the PiSy50k (c,d). (a,c) Q-Q plots comparing the p values expected based on the null distribution against the observed p values from the exact HW tests of 10 000 random SNPs on the screening array before (a) and after (c) selecting markers for the PiSy50k array. The green line indicates the expected under HW. (b,d) Ternary plots showing the genotype frequencies of 10 000 random SNPs on the screening array before (b) and after (d) selecting markers for the PiSy50k array. Blue and red dots are markers respectively following or deviating significantly from the HW expectations (Chi-square test at alpha level 0.001).

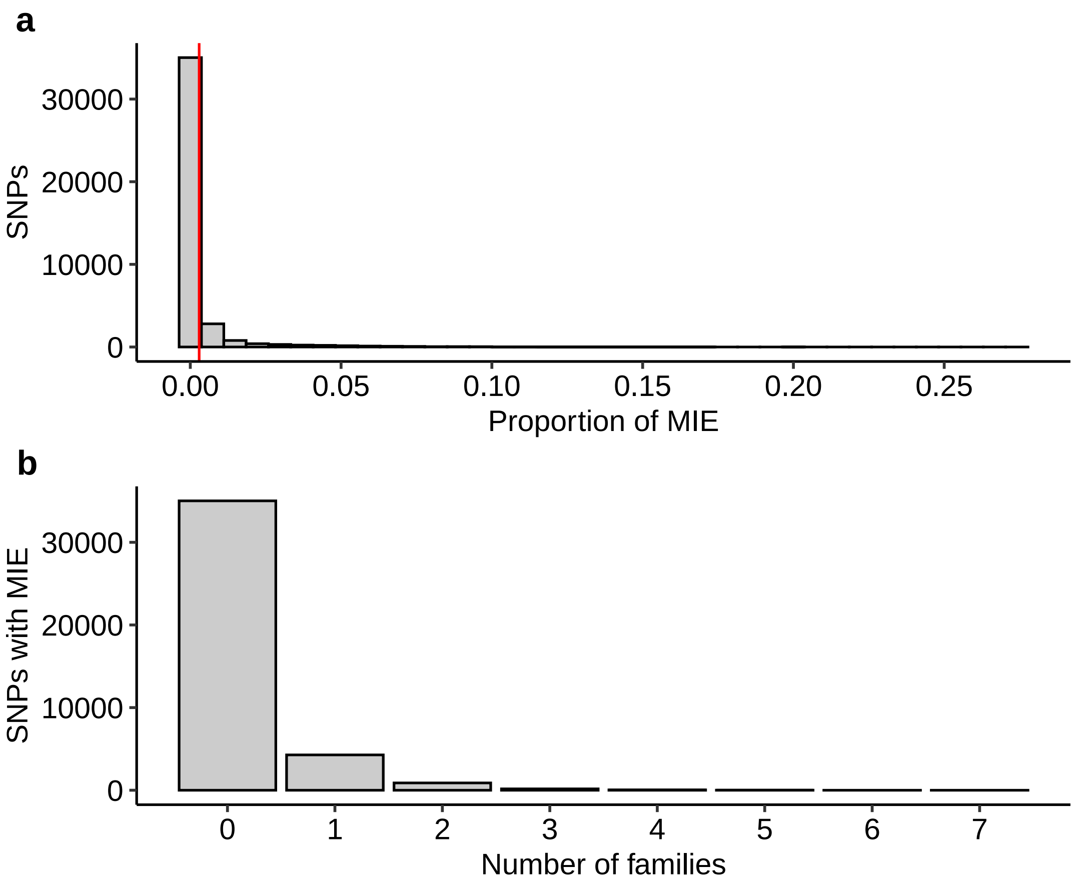

Figure S4. Mendelian errors (ME) of the PiSy50k identified in 40 405 SNPs genotyped in 135 trios (10 crosses). (a) Distribution of ME across loci, the red line indicates the mean error rate across loci (0.29%). (b) ME across families (bars at 0 and 1 indicate the number of SNPs with no ME and with ME in only one family).

2 Supporting tables

Table S1. Conversion type for markers from each data set in the screening array based on individuals with call rate 97% or above. We included the markers with the PHR and NMH conversion types (in bold) in the selection of markers for the PiSy50k array. PHR = Poly High Resolution, NMH = No Minor Homozygote, MHR = Mono High Resolution, CRBT = Call Rate Below Threshold, OTV = Off-Target Variant. Values in parenthesis are the proportion (per cent) of each conversion type in each data set.

Data set ID	Nb of markers	PHR	NMH	MHR	CRBT	Other	OTV
ProCoGen haploid	94180	16288 (17.3)	21217 (22.5)	13527 (14.4)	5901 (6.3)	1252 (1.3)	35995 (38.2)
ProCoGen diploid	23565	1395 (5.9)	7749 (32.9)	8835 (37.5)	403 (1.7)	145 (0.6)	5038 (21.4)
UOULU exomeFEB2019	25140	6486 (25.8)	1919 (7.6)	1871 (7.4)	2971 (11.8)	314 (1.2)	11579 (46.1)
UOULU RNA-seq	179240	44592 (24.9)	23658 (13.2)	39271 (21.9)	14801 (8.3)	4497 (2.5)	52421 (29.2)
UKCEH1	18901	5750 (30.4)	3130 (16.6)	8322 (44)	280 (1.5)	207 (1.1)	1212 (6.4)
UKCEH2	57261	14118 (24.7)	8765 (15.3)	12400 (21.7)	4423 (7.7)	974 (1.7)	16581 (29)
UOULU candidate	3273	1038 (31.7)	598 (18.3)	516 (15.8)	273 (8.3)	35 (1.1)	813 (24.8)
LUKE candidate	5980	251 (4.2)	371 (6.2)	3082 (51.5)	256 (4.3)	117 (2)	1903 (31.8)
Total	**407540**	**89918 (22.1)**	**67407 (16.5)**	87824 (21.5)	29308 (7.2)	7541 (1.9)	125542 (30.8)

Table S2. Number and proportions of markers from each source at different steps of the PiSy50k array design.

Data set ID	Initial set		Screening array		PiSy50k array		Screening to PiSy50k array
	count	%	count	%	count	%	%
ProCoGen haploid	1870598	48.9	94180	23.1	6995	14.7	7.4
ProCoGen diploid	304661	8	23565	5.8	340	0.7	1.4
UOULU exomeFEB2019	95504	2.5	25140	6.2	3356	7	13.3
UOULU RNA-seq	1349291	35.3	179240	44	20797	43.6	11.6
UKCEH1	20795	0.5	18901	4.6	6718	14.1	35.5
UKCEH2	175841	4.6	57261	14.1	7788	16.3	13.6
UOULU candidate	3584	0.1	3273	0.8	1187	2.5	36.3
LUKE candidate	6157	0.2	5980	1.5	531	1.1	8.9
Total	3826431	100	407540	100	47712	100	11.7

Table S3. Distribution of PiSy50k markers on *P. taeda* linkage groups (Westbrook *et al.* 2015).

Linkage Group	Length (cM)†	Nb of markers per source (ProCoGen) Haploid	Diploid	Total nb of markers count	%	average distance (cM)
1	184.89	141	3	144	8.9	3.45
2	222	120	9	129	8	3.57
3	186.88	119	4	123	7.6	3.06
4	186.32	126	8	134	8.3	3.13
5	216.41	164	5	169	10.4	2.65
6	193.57	142	6	148	9.1	2.61
7	193.43	146	11	157	9.7	2.6
8	189.56	113	5	118	7.3	2.8
9	172	126	4	130	8	2.34
10	211	136	9	145	9	2.81
11	146.89	128	7	135	8.3	2.32
12	202.48	83	4	87	5.4	3.83

†Values taken from Table 2 in Westbrook *et al.* 2015.

Table S4. Conversion type for markers from each data set in the PiSy50k array based on individuals with call rate 97% or above. We included the markers with the PHR and NMH conversion types (in bold) in further analyses. Count and proportion (%) of each conversion type is given within each data set.

Data set ID	Nb of markers on arrays	PHR count	PHR %	NMH count	NMH %	MHR count	MHR %	CRBT count	CRBT %	Other count	Other %	OTV count	OTV %
ProCoGen haploid	6995	5244	75	285	4.1	3	0	1043	14.9	418	6	2	0
ProCoGen diploid	340	255	75	19	5.6	2	0.6	39	11.5	25	7.4	0	0
UOULU exomeFEB2019	3356	2682	79.9	75	2.2	0	0	438	13.1	161	4.8	0	0
UOULU RNA-seq	20797	16788	80.7	684	3.3	4	0	2457	11.8	860	4.1	4	0
UKCEH1	6718	4871	72.5	898	13.4	652	9.7	133	2	131	1.9	33	0.5
UKCEH2	7788	6541	84	184	2.4	0	0	782	10	280	3.6	1	0
UOULU candidate	1187	614	51.7	261	22	23	1.9	164	13.8	123	10.4	2	0.2
LUKE candidate	531	132	24.9	145	27.3	43	8.1	115	21.7	92	17.3	4	0.8
Total	47712	37127	77.8	2551	5.3	727	1.5	5171	10.8	2090	4.4	46	0.1

Table S5. Evaluation of the PiSy50k array for the control samples with call rate above 97%. Values before the forward slash indicate estimates obtained from the full PiSy50k array (40 405 SNPs). Values after the forward slash indicate estimates obtained from the subset of SNPs and the needle sample also genotyped by the Axiom_PineGAP array (7 592 SNPs). CR: call rate; Het: heterozygosity. Mean pairwise error rate estimated as percentage of calls among control pairs that were different (excluding markers which had missing data in at least one of the pairs).

Tissue	Plate	CR (%)	Het (%)	Mean pairwise error rate (%)
Needle	2	98.43 / 98.13	29.17 / 24.97	1.01 / 0.50
	3	98.52 / 98.34	29.30 / 25.11	0.98 / 0.56
	7	98.38 / 98.08	29.43 / 24.95	0.99 / 0.52
Megagametophyte	4	98.66 / —	00.82 / —	0.70 / —
	2	98.34 / —	00.97 / —	0.70 / —

3 Appendix S1

Additional steps/details in selecting markers from screening array to PiSy50k array

In addition to the steps described in the main text, we performed the following filtering from the screening to the PiSy50k array. During the screening array development, we included multiple probe sets for markers of high piority to be able to select the best performing probe sets. These markers had the same Affy-SNP-ID, but differing probe set sequences. From these, there were 403 markers (806 probe sets) with the conversion type PHR or NMH. We chose the probe sets that were classified as the best probe sets by Thermo Fisher Scientific (BestProbeset = 1). We further selected the best probe sets after filtering for Mendelian errors, heterozygote errors in haploid megagametophyte samples, Hardy Weinberg equilibrium *p*-value, and minor allele frequency, but before filtering for LD. Since we analyzed the screening data before filtering, all 403 duplicate markers were included in the screening array exploration (e.g. Figures 2–3).

Additionally, since we wanted to include as many as possible markers from the previously developed Axiom PineGap array (Perry et al., 2020), we retained all markers with MAF > 0.05 and call rate > 0.8 in a previously genotyped European sample (Perry et al., 2020), as described in the main Material and Methods. During this step, in addition to markers with conversion types PHR and NMH, we also included markers with conversion type MHR on the screening array. All UKCEH1 markers (20 795 markers that performed well on the Axiom PineGAP array) were included in our screening array and shared a probe set id with our screening array markers. All UKCEH1 markers were included in this additional selection step, even if they shared a source with some of our other marker sources ("Data set ID" column of Tables S1 and S2).

We included all high-priority markers from the candidate gene sources (PacBio and UOULU candidate) and from the UKCEH1 source. As a final step to fit a maximal number of markers on the PiSy50k array, we excluded all markers that required allele-specific probes (i.e. SNPs with alleles C/G and A/T) from the low priority sources (ProCoGen haploid, ProCoGen diploid, UKCEH2 and UOULU RNA-seq). Since allele-specific probes take twice the physical space of non-allele-specific probes on the array, including only the latter allowed us to fit more markers on the PiSy50k array in total.

References

Perry, Annika, Witold Wachowiak, Alison Downing, Richard Talbot, and Stephen Cavers (2020). "Development of a Single Nucleotide Polymorphism Array for Population Genomic Studies in Four European Pine Species". In: *Molecular Ecology Resources* ().

Tajima, F. (1989). "Statistical Method for Testing the Neutral Mutation Hypothesis by DNA Polymorphism." In: *Genetics* 123.3, pp. 585–595.

Westbrook, Jared W., Vikram E. Chhatre, Le-Shin Wu, Srikar Chamala, Leandro Gomide Neves, Patricio Muñoz, Pedro J. Martínez-García, David B. Neale, Matias Kirst, Keithanne Mockaitis, C. Dana Nelson, Gary F. Peter, John M. Davis, and Craig S. Echt (2015). "A Consensus Genetic Map for Pinus Taeda and Pinus Elliottii and Extent of Linkage Disequilibrium in Two Genotype-Phenotype Discovery Populations of Pinus Taeda". In: *G3: Genes, Genomes, Genetics* 5.8, pp. 1685–1694.
